# External validation of a *red cell-based* blood prognostic score in patients with metastatic renal cell carcinoma treated with first-line immunotherapy combinations

**DOI:** 10.1007/s10585-024-10266-6

**Published:** 2024-02-16

**Authors:** Michele Maffezzoli, Matteo Santoni, Giulia Mazzaschi, Sara Rodella, Eleonora Lai, Marco Maruzzo, Umberto Basso, Davide Bimbatti, Roberto Iacovelli, Annunziato Anghelone, Ondřej Fiala, Sara Elena Rebuzzi, Giuseppe Fornarini, Cristian Lolli, Francesco Massari, Matteo Rosellini, Veronica Mollica, Cecilia Nasso, Alessandro Acunzo, Enrico Maria Silini, Federico Quaini, Massimo De Filippo, Matteo Brunelli, Giuseppe L. Banna, Pasquale Rescigno, Alessio Signori, Sebastiano Buti

**Affiliations:** 1 Department of Medicine and Surgery, University of Parma, Parma, Italy; 2 Medical Oncology Unit, University Hospital of Parma, Via Gramsci 14, 43126 Parma, Italy; 3 Oncology Unit, Macerata Hospital, 62100 Macerata, Italy; 4 Department of Oncology, Oncology Unit, Istituto Oncologico Veneto IOV IRCCS, Padua, Italy; 5 Medical Oncology, Fondazione Policlinico Universitario Agostino Gemelli IRCCS, Rome, Italy; 6 Department of Oncology and Radiotherapeutics, Faculty of Medicine and University Hospital in Pilsen, Charles University, Pilsen, Czech Republic; 7 Biomedical Centre, Faculty of Medicine in Pilsen, Charles University, Pilsen, Czech Republic; 8 Medical Oncology Unit, Ospedale San Paolo, Savona, Italy; 9 Department of Internal Medicine and Medical Specialties (Di.M.I.), University of Genova, Genoa, Italy; 10 Medical Oncology Unit 1, IRCCS Ospedale Policlinico San Martino, Genoa, Italy; 11 Department of Medical Oncology, IRCCS Istituto Romagnolo per lo Studio dei Tumori (IRST) “Dino Amadori”, Meldola, Italy; 12 Medical Oncology, IRCCS Azienda Ospedaliero-Universitaria Di Bologna, Bologna, Italy; 13 Medical Oncology, Ospedale Santa Corona, 17027 Pietra Ligure, Italy; 14 Pathology Unit, University Hospital of Parma, Parma, Italy; 15 Department of Medicine and Surgery, Section of Radiology, University of Parma, Parma, Italy; 16 Department of Diagnostic and Public Health, Section of Pathology, University of Verona, Verona, Italy; 17 Department of Oncology, Portsmouth Hospitals University NHS Trust, Portsmouth, UK; 18 Faculty of Science and Health, School of Pharmacy and Biomedical Sciences, University of Portsmouth, Portsmouth, UK; 19 Centre for Cancer, Translational and Clinical Research Institute, Newcastle University, Newcastle Upon Tyne, UK; 20 Section of biostatistics, Department of Health Sciences (DISSAL), University of Genova, Genoa, Italy

**Keywords:** Prognostic score, Blood, Mean corpuscular volume, Red cell distribution width, Metastatic renal cell carcinoma, Immunotherapy combination

## Abstract

Immunotherapy combinations with tyrosine-kinase inhibitors (TKIs) and immune checkpoint inhibitors (ICIs) had significantly improved outcomes of patients with mRCC. Predictive and prognostic factors are crucial to improve patients’ counseling and management. The present study aimed to externally validate the prognostic value of a previously developed *red cell-based score*, including hemoglobin (Hb), mean corpuscular volume (MCV) and red cell distribution width (RDW), in patients with mRCC treated with first-line immunotherapy combinations (TKI plus ICI or ICI plus ICI). We performed a sub-analysis of a multicentre retrospective observational study (ARON-1 project) involving patients with mRCC treated with first-line immunotherapy combinations. Uni- and multivariable Cox regression models were used to assess the correlation between the *red cell-based score* and progression-free survival (PFS), and overall survival (OS). Logistic regression were used to estimate the correlation between the *score* and the objective response rate (ORR). The prognostic impact of the *red cell-based score* on PFS and OS was confirmed in the whole population regardless of the immunotherapy combination used [median PFS (mPFS): 17.4 *vs* 8.2 months, HR 0.66, 95% CI 0.47–0.94; median OS (mOS): 42.0 *vs* 17.3 months, HR 0.60, 95% CI 0.39–0.92; *p* < 0.001 for both]. We validated the prognostic significance of the *red cell-based score* in patients with mRCC treated with first-line immunotherapy combinations. The *score* is easy to use in daily clinical practice and it might improve patient counselling.

**Supplementary Information:**

The online version contains supplementary material available at 10.1007/s10585-024-10266-6.

## Introduction

The treatment landscape of patients with metastatic renal cell carcinoma (mRCC) has been revolutionized by both tyrosine kinase inhibitors (TKIs) targeting the vascular endothelial growth factor receptor (VEGFR) and immune checkpoint inhibitors (ICIs). Immunotherapy combinations with TKI plus ICI and ICI plus ICI had significantly improved oncological outcomes of patients with mRCC, and represent the standard of treatment for this disease. Several combinations of TKI plus ICI and one combination of ICI plus ICI have been approved by the Food and Drugs Administration (FDA) and the European Medicines Agency (EMA) [1–5].

The choice of combination is based on the patient’s clinical features (i.e. comorbidities, performance status etc.), histological characteristics (i.e. presence of a sarcomatoid differentiation, non-clear cell component) and International mRCC Database Consortium (IMDC) risk group [6]. The latter is based on performance status (PS), time to first-line systemic therapy and other laboratory parameters (hemoglobin, neutrophil count, platelets count, serum calcium levels) [7].

Despite these therapeutic advances, predictive and prognostic factors are largely lacking. Reliable biomarkers based on the underlying disease mechanisms and drugs pharmacodynamics should ideally guide clinical decision making to select the appropriate combination [8–10].

RCC development and progression is largely sustained by the hypoxia-inducible factor-1 alpha (HIF-1α) downstream pathway, which plays a key role in metabolic adaptation, angiogenesis, cell growth, differentiation and survival [11, 12]. HIF-1α is an oxygen-sensitive subunit activated during hypoxia, which allows the heterodimerization with the other subunit, HIF-1β [11, 12]. This process leads to the activation of the HIF-1 transcriptional complex, which is responsible for the transcription of over 100 genes [11, 12]. In normoxic conditions, Von Hippel-Lindau protein (pVHL) is involved in the proteasomal degradation of HIF-1α [11, 12]. The loss of the VHL oncosuppressor gene is frequent in clear-cell RCCs (ccRCCs) and leads to upregulation of HIF-1α expression and its downstream pathway, including VEGFR axis, which promotes aberrant angiogenesis [11, 12]. Hence, VEGFR-TKIs inhibiting this signaling cascade emerged as a frontline treatment in ccRCC [12].

The pseudo-hypoxic state caused by HIF-1 pathway activation could also increase red blood-cell (RBC), stimulating erythropoietin expression [12]. On the other hand, anaemia is a common condition in patients with mRCC and has a detrimental effect on survival, according to both the IMDC and Memorial Sloan Kettering Cancer Centre (MSKCC) score [7, 13]. Anaemia is also one of the most common side effects of VEGFR-TKIs, although an increased hemoglobin (Hb) concentration and RBC count has also been noted after treatment with these agents [14–17]. Yet, TKIs could modify other RBC parameters such as mean corpuscular volume (MCV) and red cell distribution width (RDW), which reflects anisocytosis [14, 18–22].

Our previous study showed that a significant proportion of patients with mRCC undergoing TKIs (pazopanib or cabozantinib) exhibited an increased MCV and/or RDW at baseline. A higher MCV (macrocytosis) at baseline was associated with improved PFS in patients treated with pazopanib, while a higher RDW (anisocytosis) was linked to a poorer prognosis in all patients who received pazopanib or cabozantinib [14]. Hence, macrocytosis, lower degree of anisocytosis and higher level of Hb were found to be positive prognostic factors. Focusing on the same population, our group developed a *red cell-based score* through the integration of Hb, MCV and RDW, and delineated two prognostic groups: unfavourable group (0–1 good factors) and favourable group (2–3 good factors). Irrespective of other established prognostic factors, patients in the favourable group demonstrated significantly longer PFS and OS when compared to those in the unfavourable group [23]. In addition to the prognostic significance, these studies suggested that Hb, MCV and RDW may serve as an indirect indicators of the activation and alterations of the HIF-1α pathway among patients with mRCC undergoing TKIs [24].

The present sub-analysis aimed to validate the *red cell-based score* and evaluate its prognostic significance in a more contemporary cohort of patients who underwent first-line treatment with immunotherapy combinations. Furthermore, we explored whether the prognostic *score* might perform differently among patients treated with TKI plus ICI *vs* ICI plus ICI. Finally, the value of the *score* was challenged in terms of response to treatment.

## Materials and methods

This sub-analysis of a multicentre observational retrospective study was conducted on patients with mRCC undergoing first-line immunotherapy combinations – TKI plus ICI or ICI plus ICI (i.e. ipilimumab plus nivolumab) – between January 2016 and December 2022 in ten centres. The *red cell-based score* was based on the integration of Hb, MCV and RDW values. According to our previous study on patients with mRCC treated with TKIs, MCV > 87 fl (macrocytosis), RDW $\le$ 16% (anisocytosis) and Hb ≥ 12 g/dL (absence of anaemia) were considered favourable prognostic factors. Based on the number of positive prognostic factors, we divided our patient population into two groups: favourable group (2–3 good factors) and unfavourable (0–1 good factors) [23].

In the present study, VEGFR inhibitors and the anti-VEGF monoclonal antibody bevacizumab were defined as TKIs.

Patients in the study cohort had histologically proven unresectable or mRCC. They received a first-line immunotherapy combination, including avelumab plus axitinib, pembrolizumab plus axitinib, ipilimumab plus nivolumab, atezolizumab plus bevacizumab, cabozantinib plus nivolumab, lenvatinib plus pembrolizumab.

We collected the following baseline (before the beginning of first-line treatment) data: clinicopathological records (i.e. sex, smoking habit, IMDC score), surgical treatment of the primary tumour, metastatic involvement, presence of metastases at diagnosis, histopathological characteristics, and haematological-biochemical parameters including MCV, RDW and Hb levels. Systemic treatments other than those described above, and a lack of medical records were exclusion criteria.

The study was conducted following the approval by the ethics committee of the coordinating Centre (Comitato Etico Regionale delle Marche, ARON-1 study, NCT05287464, date of approval: April 21, 2022) and then by the ethics committee of each participating centre. The obtainment of informed consent for live patients was mandatory. The present study is a sub-analysis of the ARON-1 study that was designed for globally analysed real-world data from patients with mRCC receiving immunotherapy combinations.

Patient characteristics were delineated by descriptive statistics. Objective response rate (ORR) was defined as the sum of complete responses (CR) and partial responses (PR) assessed in each centre according to Response Evaluation Criteria in Solid Tumours (RECIST version 1.1). Stable disease (SD) and progressive disease (PD) were assessed by referring to the same criteria. PFS was defined as the time from the beginning of the immunotherapy combination therapy and the progression of the disease or death, whichever occurred first. The OS was intended as the time between treatment initiation and death for any reason. Patients were considered censored if they were free from progression or alive at the last follow-up. Of the 398 patients enrolled in the study, 36 (8%) were not included in the statistical analysis due to missing data.

The Kaplan–Meier method was used to estimate progression-free survival (PFS) and overall survival (OS), and the log-rank test (Mantel-Cox) was applied to assess whether there were statistically significant differences in PFS and OS across subgroups. Univariable and multivariable Cox proportional hazards regression models were used to analyse the PFS and OS data. An interaction test was performed to examine whether the *red cell-based score* had a significantly different prognostic impact in PFS and OS between patients treated with TKI + ICI and those treated with ICI + ICI. The results were expressed as Hazard Ratio (HR), 95% confidence intervals (95%CI), and *p* values. The univariable model was fitted including the following covariates known to be robust prognostic factors for patients with mRCC: sex, smoking habit, surgery, histology, sarcomatoid differentiation, presence of synchronous metastases at diagnosis, lung metastases, bone metastases, liver metastases, brain metastases, number of sites involved in the tumour, IMDC risk group, the type of combination therapy and body mass index (BMI). The multivariable model was subsequently developed taking into account only those variables that were significant at the univariable analysis.

To compare categorical endpoints Pearson’s chi-square test or Fisher’s exact test was used and the effect was expressed as Odd Ratio (OR). The level of statistical significance was set to a value of 0.05. Logistic regression were used to assess the correlation between the *score* and the ORR.

For the multivariable prognostic model (*red cell-based score*) the discriminatory ability as defined by Harrel’s c-index was calculated, both for PFS and OS (a higher c-index represented a better capability of the multivariable model to separate patients with and without the event).

The software JAMOVI version 2.3.21 (www.jamovi.org) was used to perform all the computational analyses and to draw the survival curves.

## Results

A total of 398 patients with mRCC were enrolled during the study period. Baseline clinicopathological characteristics of the overall population were reported in supplementary files (Supplementary Table 1). A significant prevalence of males over females was noted (74% *vs* 26%). The median age was 66 years (IQR 57–74): 64% were under 70 and 36% were over 70 years. The mean patient BMI value was 25.4 kg/m^2^ (range 14.7–45.3); nearly 16% of patients were obese with a BMI greater than 30 kg/m^2^.

Clear cell was the most representative histotype and accounted for 351 patients (89%). Papillary and chromophobic types accounted for 19 and 4 patients (4.5% and 1%), respectively. In 4% of patients, the histology was otherwise not specified. In 11% of cases a sarcomatoid differentiation was reported. Nephrectomy was performed in 240 (66%) patients, out of which 232 (58%) underwent a radical nephrectomy.

Patients with synchronous metastatic disease at diagnosis were 213 (53.5%), while 185 (46.5%) patients had metachronous metastatic lesions. The most involved site was the lung (68%), followed by the abdominal lymph nodes (39%), bones (33%) and mediastinal lymph nodes (32%), while the liver (16%), brain (9%) and soft tissues (13%) were less involved. Only 20% of patients had more than three localizations, while 77% had three or fewer metastatic sites.

According to the IMDC criteria, 264 (66%) of patients belonged to the intermediate prognostic group, while 14% and 20% were in the good- and poor-risk groups, respectively.

First line treatment consisted of ICI plus ICI—ipilimumab plus nivolumab—in 150 (38%) patients, and TKI plus ICI in 248 patients (62%). The most commonly used combination was pembrolizumab plus axitinib (47%). At a median follow-up time of 16.1 months (95%CI 14.3–18.8), 41% of patients were still receiving the first-line treatment.

Six patients (2%) achieved a CR, 173 (44%) PR and 105 (26%) had SD, while 80 patients (20%) had PD as the best response. PD occurred in 190 (48%) patients and 134 (34%) died.

The median PFS (mPFS) of the overall population was 14.7 months (95%CI 12.2–18.9) with a total of 208 censored patients (Supplementary Fig. 1a). The median OS was 33.3 months (95%CI 26.1-not calculated), with 134 deceased cases (Supplementary Fig. 1b).

The study population characteristics according to the *red cell-based score* were reported in Table 1. Unfavourable group (0–1 good factors) accounted for 117 patients (32%), while 245 (68%) were in the favourable group (2–3 good factors). The groups were significantly unbalanced (*p* < 0.05) for the following features: sex (Supplementary Fig. 2a), surgery, lung metastases, IMDC group and BMI. Particularly, only 54% of patients in the unfavourable group had received nephrectomy (Supplementary Fig. 2b), compared to 72% of patients in the favourable one (*p* < 0.001). Lung metastases were more common in the unfavourable than in the favourable group (*p* = 0.046) (Supplementary Fig. 2c). There was an imbalance in the distribution of the IMDC score among the *red cell-based score* groups. In particular, 38% of patients in the unfavourable group were classified as poor risk and only 11% in the favourable group (*p* < 0.001) (Supplementary Fig. 2d). In addition, patients in the favourable group had a higher BMI (*p* = 0.005) (Supplementary Fig. 2e). Furthermore, the IMDC prognostic categories distribution was significantly different within the *red cell-based score* groups when patients were stratified according to the type of immunotherapy combination used (*p* < 0.001) (Supplementary Fig. 3, Table 2).

**Table 1:** Population characteristics according to the red cell-based score

	0–1good factor (group 1: unfavourable) (%)	2–3 good factors (group 2: favourable) (%)	Total (%)	p-value
Number of patients	117 (32)	245 (68)	362 (100)	
Sex				**0.040**
Male	80 (68)	192 (78)	272 (75)	
Female	37 (32)	53 (22)	90 (25)	
Current or formers smoker				
Missing	3 (3)	6 (2)	9 (2)	
No	84 (72)	153 (62)	237 (65)	
Yes	30 (25)	86 (36)	116 (33)	0.071
Surgery				** < .001**
No	54 (46)	68 (28)	122 (34)	
Yes	63 (54)	177 (72)	240 (66)	
Histology				0.231
Missing	2 (2)	4 (2)	6 (2)	
Clear cell	105 (89)	217 (89)	322 (89)	
Papillary	2 (2)	14 (5)	16 (4)	
Chromophobe	1 (< 1)	2 (< 1)	3 (< 1)	
Other	7 (6)	8 (3)	15 (4)	
Sarcomatoid differentiation				0.057
Missing	11 (9)	9 (4)	20 (6)	
No	88 (75)	213 (87)	301 (83)	
Yes	18 (16)	23 (9)	41 (11)	
Lung metastases				**0.046**
No	28 (24)	84 (34)	112 (31)	
Yes	89 (76)	161 (66)	259 (69)	
Bone metastases				0.138
No	72 (62)	170 (69)	242 (67)	
Yes	45 (38)	75 (31)	120 (33)	
Brain metastases				0.362
No	140 (89)	225 (92)	329 (91)	
Yes	13 (11)	20 (8)	33 (9)	
Liver metastases				0.319
No	95 (81)	209 (85)	304 (84)	
Yes	22 (19)	36 (15)	58 (16)	
Number of sites				0.061
$\le$ 3 sites	86 (74)	201 (82)	287 (79)	
$>$ 3 sites	31 (26)	44 (18)	75 (21)	
IMDC group				** < .001**
Good	8 (7)	45 (18)	53 (15)	
Intermediate	65 (56)	174 (71)	239 (66)	
Poor	44 (38)	26 (11)	70 (19)	
Combination type				0.809
TKI + ICI	72 (62)	154 (63)	226 (62)	
ICI + ICI	45 (38)	91 (37)	136 (38)	
Best response				0.328
CR	0 (0)	4 (1)	4 (1)	
PR	52 (45)	107 (44)	159 (44)	
SD	33 (28)	68 (28)	101 (28)	
PD	26 (22)	43 (18)	69 (19)	
NV	6 (5)	23 (9)	29 (8)	
Age				0.100
Median	65	66	66	
CI 95	57–71	58–74	27–89	
BMI				**0.005**
Missing	0	1.0	1.0	
Mean (SD)	24.7 (4.0)	26.1 (4.4)	25.7 (4.3)	
Range	17.8–39.2	14.7–45.3	14.7–45.3	

*IMDC score* International Metastatic RCC Database Consortium Score; *TKI* Tyrosine-Kinase Inhibitor; *ICI* Immune Checkpoint Inhibitor; *CR* Complete Response; *PR* Partial Response; *SD* Stable Disease; *PD* Progression Disease; *BMI* Body Mass Index. *NV* not valuable

Bold indicates statistically significant values

**Fig. 1:**
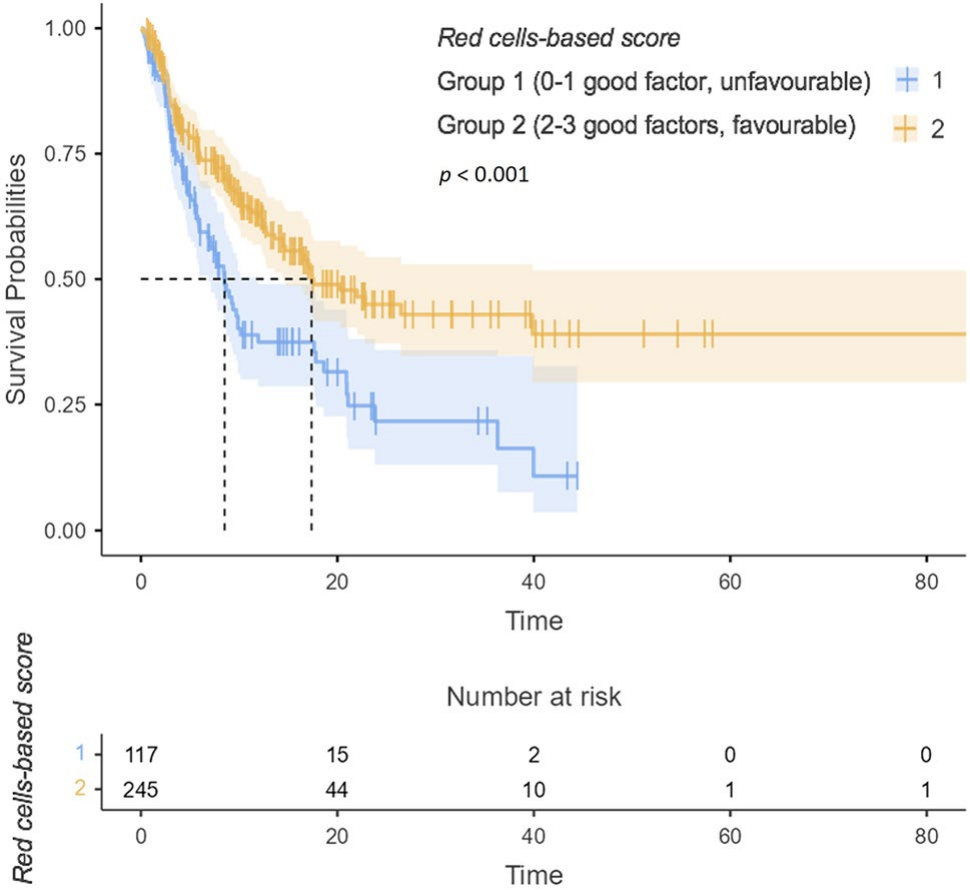
Representative Kaplan–Meier survival curve illustrating the impact of the red cell-based score on PFS

Regarding the PFS, primary tumour in site (no surgery for primary tumour), presence of bone or brain metastases, more than three metastatic sites, poor IMDC risk group, and therapy with ipilimumab plus nivolumab were significantly associated with shorter PFS at univariable analysis. The favourable *red cell-based score* was associated with longer PFS (HR 0.54, 95%CI 0.39–0.74,* p* < 0.001). When challenged in the multivariable model, more than three metastatic sites, TKI plus ICI combination and favourable *red cell-based score* (HR 0.63, 95%CI 0.45–0.88,* p* = 0.008) confirmed their positive prognostic value in terms of PFS. (Table 2 and Supplementary Fig. 4). The mPFS was 8.5 months (95%CI 6.8–11.9) for the 117 patients in the unfavourable group, and 17.4 months (95%CI 14.5-not estimable) for the 245 patients favourable one (Fig. 1). The accuracy of the score (*c*-index) for PFS was 0.57.

**Table 2:** Explanatory prognostic factors of PFS in uni- and multivariable Cox proportional hazard models

PFS	All 332 (100%)	Univariable			Multivariable		
		HR	95%CI	*p*	HR	95%CI	*p*
Sex				0.059			
Male	249 (75%)	Reference					
Female	83 (25%)	1.38	0.99–1.92				
Current or formers smokers				0.814			
No	224 (68%)	Reference					
Yes	108 (32%)	0.96	0.69–1.34				
Surgery				** < 0.001**			0.111
No	104 (31%)	Reference			Reference		
Yes	228 (69%)	0.55	0.40–0.76		0.75	0.53–1.07	
Clear cell				0.939			
No	34 (10%)	Reference					
Yes	298 (90%)	1.02	0.62–1.69				
Sarcomatoid differentiation				0.323			
No	294 (89%)	Reference					
Yes	38 (11%)	1.26	0.80–1.99				
Synchronous metastatic disease at diagnosis				0.051			
No	156 (47%)	Reference					
Yes	176 (53%)	1.37	1.00–1.87				
Lung metastases				0.375			
No	100 (30%)	Reference					
Yes	232 (70%)	1.17	0.83–1.65				
Bone metastases				**0.002**			0.254
No	227 (68%)	Reference			Reference		
Yes	105 (32%)	1.66	1.20–2.28		1.24	0.86–1.78	
Liver metastases				0.059			
No	281 (85%)	Reference					
Yes	51 (15%)	1.47	0.98–2.20				
Brain metastases				**0.006**			0.106
No	302 (91%)	Reference			Reference		
Yes	30 (9%)	1.90	1.20–3.01		1.50	0.92–2.44	
Number of sites				** < 0.001**			**0.049**
$\le$ 3 sites	268 (81%)	Reference			Reference		
$>$ 3 sites	64 (19%)	2.12	1.49–3.02		1.53	1.00–2.34	
IMDC group							
Good	47 (14%)	Reference			Reference		
Intermediate	220 (66%)	1.63	0.95–2.80	0.079	1.14	0.63–2.03	0.668
Poor	65 (20%)	2.91	1.60–5.27	** < 0.001**	1.50	0.76–2.95	0.240
Combination type				**0.031**			**0.040**
TKI + ICI	203 (61%)	Reference			Reference		
ICI + ICI	129 (39%)	1.41	1.03–1.93		1.42	1.02–1.99	
BMI				0.061			
Mean (SD)	25.8 (4.3)	0.96	0.93–1.00				
AGE				0.351			
Mean (SD)	64.5 (11.0)	1.01	0.99–1.02				
Red cell-based score				** < 0.001**			**0.008**
Group 1	103 (31%)	Reference			Reference		
Group 2	229 (69%)	0.54	0.39–0.74		0.63	0.45–0.88	

*Ref* Reference; *HR* Hazard Ratio; *95%CI* 95% Confidence Intervals; *PFS* Progression Free Survival; *IMDC* International mRCC Database Consortium score; *TKI* Tyrosine-Kinase Inhibitor; *ICI* Immune Checkpoint Inhibitor; *BMI* Body Mass Index

Red cell-based score: group 1: unfavourable; group 2: favourable

Bold indicates statistically significant values

**Fig. 2:**
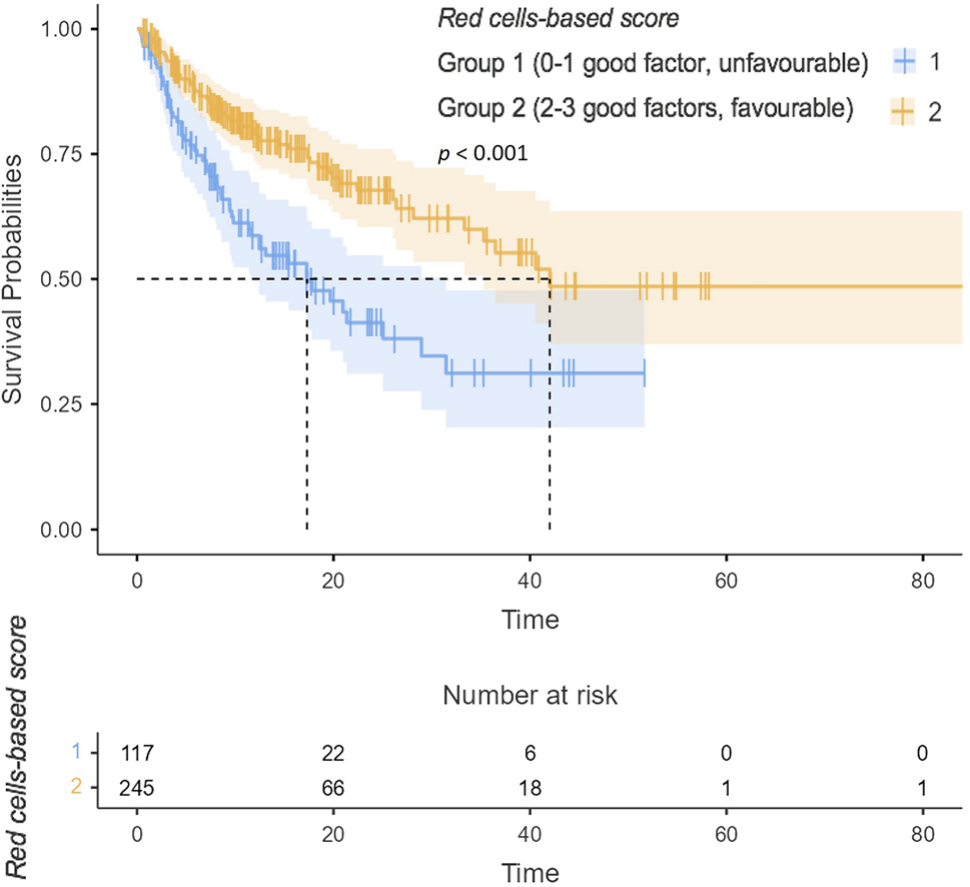
Representative Kaplan–Meier survival curve illustrating the impact of the red cell-based score on OS

Concerning the OS, primary tumour in site (no surgery for primary tumour), synchronous metastatic disease at diagnosis, presence of bone, liver or brain metastases, more than three metastatic sites, IMDC poor-risk group and higher BMI were also significantly associated with shorter OS at univariable analysis. Favourable *red cell-based score* was associated with longer OS (HR 0.46, 95%CI 0.32–0.67,* p* < 0.001). The absence of brain metastases, higher BMI and favourable *red cell-based score* (HR 0.62, 95%CI 0.41–0.93,* p* = 0.021), all demonstrated their favourable prognostic value in terms of OS in the multivariable model (Table 3 and Supplementary Fig. 5). Patients in the favourable group had significantly longer mOS (42.0 months, 95%CI 35.3-not estimable) when compared with the unfavourable one (17.3 months, 95%CI 11.6–31.4) (Fig. 2). The accuracy of the score (c-index) in terms of OS was 0.60.

**Table 3:** Explanatory prognostic factors of OS in uni- and multivariable Cox proportional hazard models

OS	All 332 (100%)	Univariable			Multivariable		
		HR	95%CI	*p*	HR	95%CI	*p*
Sex				0.959			
Male	249 (75%)	Reference					
Female	83 (25%)	1.01	0.66–1.54				
Current or formers smokers				0.565			
No	224 (68%)	Reference					
Yes	108 (32%)	1.12	0.76–1.66				
Surgery				**0.001**			0.194
No	104 (31%)	Reference			Reference		
Yes	228 (69%)	0.52	0.35–0.76		0.73	0.45–1.18	
Clear cell				0.766			
No	34 (10%)	Reference					
Yes	298 (90%)	0.92	0.51–1.63				
Sarcomatoid differentiation				0.066			
No	294 (89%)	Reference					
Yes	38 (11%)	1.61	0.97–2.66				
Synchronous metastatic disease at diagnosis				**0.019**			0.548
No	156 (47%)	Reference			Reference		
Yes	176 (53%)	1.58	1.08–2.33		0.86	0.52–1.42	
Lung metastases				0.446			
No	100 (30%)	Reference					
Yes	232 (70%)	1.18	0.77–1.80				
Bone metastases				**0.003**			0.406
No	227 (68%)	Reference			Reference		
Yes	105 (32%)	1.79	1.22–2.62		1.21	0.77–1.90	
Liver metastases				**0.014**			0.271
No	281 (85%)	Reference			Reference		
Yes	51 (15%)	1.79	1.13–2.84		1.32	0.80–2.18	
Brain metastases				** < 0.001**			**0.013**
No	302 (91%)	Reference			Reference		
Yes	30 (9%)	2.60	1.56–4.31		2.02	1.16–3.54	
Number of sites				** < 0.001**			0.172
$\le$ 3 sites	268 (81%)	Reference			Reference		
$>$ 3 sites	64 (19%)	2.47	1.64–3.73		1.45	0.85–2.48	
IMDC group							
Good	47 (14%)	Reference			Reference		
Intermediate	220 (66%)	1.54	0.76–3.09	0.229	1.18	0.56–2.15	0.669
Poor	65 (20%)	3.40	1.65–7.26	**0.001**	1.95	0.84–4.53	0.122
Combination type				0.270			
TKI + ICI	203 (61%)	Reference					
ICI + ICI	129 (39%)	1.24	0.85–1.82				
BMI				**0.012**			**0.030**
Mean (SD)	25.8 (4.3)	0.94	0.90–0.99		0.94	0.90–0.99	
AGE				0.200			
Mean (SD)	64.5 (11.0)	1.01	0.99–1.03				
Red cell-based score				** < 0.001**			**0.021**
Group 1	103 (31%)	Reference			Reference		
Group 2	229 (69%)	0.46	0.32–0.67		0.62	0.41–0.93	

*Ref* reference; *HR* Hazard Ratio; *95%CI* 95% Confidence Intervals; *OS* Overall Survival; *IMDC* International mRCC Database Consortium score; *TKI* Tyrosine-Kinase Inhibitor; *ICI* Immune Checkpoint Inhibitor; *BMI* Body Mass Index

Red cell-based score: group 1: unfavourable; group 2: favourable

Bold indicates statistically significant values

Therefore, the *red cell-based score* retained a statistically significant prognostic impact on both PFS and OS.

Regarding ORR, clear cell histology, presence of bone metastases, more than three metastatic sites, and therapy with ipilimumab plus nivolumab resulted negative predictors of response to treatment at univariable analysis (Table 4**).** The multivariable regression model showed a significant association between a lower ORR and presence of bone metastases (*p* = 0.044), ICI plus ICI combination (*p* = 0.001), and with more than three metastatic sites (*p* = 0.020). No significant association was observed between the *red cell-based score* and ORR (Table 4 and Supplementary Fig. 6).

**Table 4:** Logistic regression model for objective response rate

Response	Responder		Univariable			Multivariable		
	No	Yes	OR	95%CI	*p*	OR	95%CI	*p*
Sex					0.165			
Male	133 (49%)	140 (51%)	Reference					
Female	52 (57%)	39 (43%)	0.71	0.44–1.15				
Current or formers smokers					0.259			
No	129 (53%)	115 (47%)	Reference					
Yes	52 (46%)	60 (54%)	1.29	0.83–2.03				
Surgery					0.268			
No	67 (55%)	55 (45%)	Reference					
Yes	118 (49%)	124 (51%)	1.28	0.83–1.99				
Clear cell					**0.049**			0.057
No	28 (65%)	15 (35%)	Reference			Reference		
Yes	157 (49%)	164 (51%)	1.95	1.02–3.87		1.96	0.99–4.01	
Sarcomatoid differentiation					0.383			
No	157 (51%)	149 (49%)	Reference					
Yes	19 (44%)	24 (56%)	1.33	0.70–2.56				
Synchronous metastatic disease at diagnosis					0.936			
No	85 (51%)	83 (49%)	Reference					
Yes	100 (51%)	96 (49%)	0.98	0.65–1.49				
Lung metastases					0.367			
No	64 (54%)	54 (46%)	Reference					
Yes	121 (49%)	125 (51%)	1.22	0.79–1.90				
Bone metastases					**0.003**			**0.044**
No	115 (46%)	137 (54%)	Reference			Reference		
Yes	70 (62%)	42 (38%)	0.50	0.32–0.79		0.61	0.37–0.99	
Liver metastases					0.155			
No	150 (49%)	155 (51%)	Reference					
Yes	35 (59%)	24 (41%)	0.66	0.37–1.16				
Brain metastases					0.226			
No	166 (50%)	167 (50%)	Reference					
Yes	19 (61%)	12 (39%)	0.63	0.29–1.32				
Number of sites					**0.003**			**0.020**
$\le$ 3 sites	136 (47%)	154 (53%)	Reference			Reference		
$>$ 3 sites	49 (66%)	25 (34%)	0.45	0.26–0.76		0.51	0.28–0.89	
IMDC group								
Good	23 (45%)	28 (55%)	Reference					
Intermediate	119 (49%)	122 (51%)	0.84	0.46–1.54	0.579			
Poor	43 (60%)	29 (40%)	0.55	0.27–1.14	0.110			
Combination type					**0.001**			**0.001**
TKI + ICI	102 (44%)	128 (56%)	Reference			Reference		
ICI + ICI	83 (62%)	51 (38%)	0.49	0.32–0.75		0.46	0.29–0.71	
BMI					0.484			
Mean (SD)	25.5 (4.4)	25.8 (4.3)	1.02	0.97–1.07				
Red cell-based score					0.587			
Group 1	59 (53%)	52 (47%)	Reference					
Group 2	111 (50%)	111 (50%)	1.13	0.72–1.79				

*Ref* reference; *OR* Odds Ratio; *95%CI* 95% Confidence Intervals; *IMDC* International mRCC Database Consortium score; *TKI* Tyrosine-Kinase Inhibitor; *ICI* Immune Checkpoint Inhibitor; *BMI* Body Mass Index

Red cell-based score: group 1: unfavourable; group 2: favourable

Bold indicates statistically significant values

As shown in Supplementary Fig. 7, the *red cell-based score* was able to hold its prognostic value in terms of PFS, regardless of the combination treatment (*p* < 0.0001, log-rank test). The HR was 0.60 (95%CI 0.37–0.95) for TKI plus ICI (0.52 excluding IMDC good-risk patients, 95%CI 0.33–0.80) and 0.51 (95%CI 0.34–0.77) for ICI plus ICI. No significant interaction was detected between the type of immunotherapy combination used and the *red cell-based score* group in terms of PFS (*p* = 0.64; *p* = 0.66 excluding good-risk patients).

The *red cell-based score* also demonstrated a good prognostic performance in terms of OS, regardless of the combination treatment (*p* < 0.0001, log-rank test). The HR for the TKI plus ICI group was 0.45 (95%CI 0.27–0.73; 0.51 without considering IMDC good-risk patients, 95%CI 0.30–0.86), and 0.49 (95%CI 0.29–0.85) for ICI plus ICI group (Supplementary Fig. 8). We did not observe a significant interaction between the type of immunotherapy combination used and the *red cell-based score* group (*p* = 0.94; *p* = 0.86 without considering IMDC good-risk patients).

Finally, the *score* was not able to predict the response to cancer treatment (CR and PR *vs* non-responders), irrespective of the type of immunotherapy combination administered.

## Discussion

Different combinations of TKIs and ICIs are currently approved as first-line treatment of patients with mRCC [1–5]. The choice of the combination is mostly based on clinical and histological features [6]. However, only a portion of patients with mRCC can gain a meaningful benefit from these therapeutic approches. Prediction of response to treatment and counselling regarding patients’ prognosis remains a challenge. Thus, the identification of clinical and laboratory features endowed with prognostic or predictive potential in daily practice might significantly improve patient management [8–10, 25].

Among readily available laboratory parameters, anaemia is widely acknowledged as a significant negative prognostic factor, thereby it has been included in both the MSKCC and IMDC prognostic scores [7, 13]. Indeed, Hb level below the lower limit of normal is associated with shorter OS and PFS [26–29]. It has been demonstrated that the use of TKIs causes significant alterations in Hb levels [14, 28], nevertheless, the prognostic significance of these changes is still debated [30]. Several studies showed that the occurrence of an increased Hb level during TKI treatment may be related to longer survival. [15–17]. Moreover, it has been consistently noted that TKI therapy is associated with MCV and RDW changes [14, 18]. Macrocytosis at baseline and following TKI treatment has been correlated to a better survival outcome [14, 19–22]. Other studies documented a correlation between RDW and the outcome of patients with mRCC as higher RDW at baseline, which reflects anisocytosis, has been associated with a poorer PFS and OS [14, 31, 32]. So, macrocytosis, lower degree of anisocytosis and higher level of Hb were found to be positive prognostic factors in patients treated with TKIs [14]. Accordingly, based on these observations we are planning a prospective study to understand the mechanistic basis underlying the changes in the red cell parameters during the treatment with TKIs.

Our previous study demonstrated the prognostic significance of the *red cell-based score* incorporating Hb, MCV, and RDW, in patients with mRCC undergoing TKI treatment. Patients carrying at least two favourable prognostic factors experienced notably extended PFS and OS compared to those with 0 to 1 positive prognostic factors [23]. In the present work, we aimed to validate the *red cell-based score* in a more actual clinical setting involving a population of patients with mRCC treated with first-line immunotherapy combinations (TKI plus ICI or ICI plus ICI). According to our previous data, patients with at least two favourable prognostic features exhibited significantly longer PFS and OS than patients belonging to the unfavourable group (0 to 1 positive prognostic factors), regardless of the combination used. Notably, the *red cell-based score* maintained its prognostic significance in terms of both PFS and OS at multivariable analysis, when adjusted for several clinical-pathological features known as reliable prognostic factors for patients with mRCC, including the IMDC score. However, no significant interaction was detected between the type of immunotherapy combination used and the *red cell-based score* group, when considering PFS, OS and ORR. Instead, the *score* failed to demonstrate a prediction of tumour response.

The laboratory parameters included in the *score* are inexpensive and easy to use in daily clinical practice. Our prognostic model is based on real-world patients, thus it might improve counselling and selection of patients who might benefit most from treatments.

Limitations of the present study might reside in its retrospective design which may have resulted in selection bias and data collection bias. Moreover, due to the relatively short follow-up (high number of censored patients in the first part of the curves), the survival curves were not mature enough to establish the mOS in all groups. Finally, each centre independently managed the treatment, and assessed the response to therapy (based on RECIST 1.1) according to the local clinical practice.

## Conclusion

The prognostic value of our *red cell-based score* is validated in a wide contemporary series of patients with mRCC. The *score* maintains its prognostic value regardless of the type of first-line immunotherapy combination therapy and irrespective of the IMDC score. The laboratory-based biomarkers included in the *score* are inexpensive and easy to look in clinical practice. It could give to the clinicians more information regarding patients’ prognosis.

This prognostic tool can also be validated in other therapy settings, such as second- or further-line therapy. Future studies are warranted to prospectively validate our *score* and to understand why the RBC parameters are strongly related to prognosis, irrespective of the treatment received.

### Supplementary Information

Below is the link to the electronic supplementary material.

Supplementary file1 (DOCX 51675 KB)

## Data Availability

All data generated or analysed during this study are included in this published article and its supplementary information files.
