# Supplementary material for: External validation of a red cell-based blood prognostic score in patients with metastatic renal cell carcinoma treated with first-line immunotherapy combinations
Source: Clin Exp Metastasis. 2024 Feb 16;41(2):117–29. doi: 10.1007/s10585-024-10266-6 (PMC10973030; doi:10.1007/s10585-024-10266-6)
Supplement: Supplementary file 1 — Supplementary file1 (DOCX 51675 KB) [file 10585_2024_10266_MOESM1_ESM.docx]

**Supplementary Table 1** Clinicopathological characteristics

|  | **n** |
| --- | --- |
| ***Overall Patients Population*** | 398 (100%) |
| ***Sex*** |  |
| Male | 296 (74.4%) |
| Female | 102 (25.6%) |
| ***Histology*** |  |
| Clear cell | 351 (88.7%) |
| Papillary | 19 (4.5%) |
| Chromophobe | 4 (1.0%) |
| Other specify | 17 (4.3%) |
| Missing | 7 (1.7%) |
| ***Sarcomatoid differentiation*** |  |
| No | 332 (83.4%) |
| Yes | 44 (11%) |
| Missing | 22 (5.5%) |
| ***Grading*** |  |
| 1 | 12 (3.0%) |
| 2 | 67 (16.8%) |
| 3 | 98 (24.6%) |
| 4 | 99 (24.9%) |
| Missing | 122 (30.7%) |
| ***Surgery*** |  |
| No | 136 (34.2%) |
| Yes | 262 (65.8%) |
| ***Type of surgery*** |  |
| Total nephrectomy | 232 (58.3%) |
| Partial nephrectomy | 27 (6.8%) |
| Missing | 139 (34.9%) |
| ***Synchronous metastatic disease at diagnosis*** |  |
| No | 185 (46.5%) |
| Yes | 213 (53.5%) |
| ***Metastatic sites*** |  |
| ***Lung*** |  |
| No | 128 (32.3%) |
| Yes | 270 (67.8%) |
| ***Bone*** |  |
| No | 265 (66.6%) |
| Yes | 133 (33.4%) |
| ***Liver*** |  |
| No | 334 (83.9%) |
| Yes | 64 (16.1%) |
| ***Brain*** |  |
| No | 363 (91.2%) |
| Yes | 35 (8.8%) |
| ***Mediastinal lymph nodes*** |  |
| No | 272 (68.3%) |
| Yes | 126 (31.7%) |
| ***Abdominal lymph nodes*** |  |
| No | 244 (61.3%) |
| Yes | 154 (38.7%) |
| ***Soft tissue*** |  |
| No | 346 (86.9%) |
| Yes | 52 (13.1%) |
| ***Other sites*** |  |
| No | 94 (23.6%) |
| Yes | 131 (32.9%) |
| Missing | 173 (43.5%) |
| ***IMDC group*** |  |
| Good | 54 (13.6%) |
| Intermediate | 264 (66.3%) |
| Poor | 80 (20.1%) |
| ***Drugs combination*** |  |
| Pembrolizumab + Axitinib | 188 (47.2%) |
| Avelumab + Axitinib | 26 (6.5%) |
| Ipilimumab + Nivolumab | 150 (37.7%) |
| Other | 34 (8.5%) |
| ***Combination type*** |  |
| TKI + ICI | 248 (62.3%) |
| ICI + ICI | 150 (37.7%) |

*IMDC score: International Metastatic RCC Database Consortium Score; TKI: Tyrosine-Kinase Inhibitor; ICI: Immune Checkpoint Inhibitor.*

**Supplementary Fig. 1a** Survival curve based on Kaplan-Meier estimates of PFS


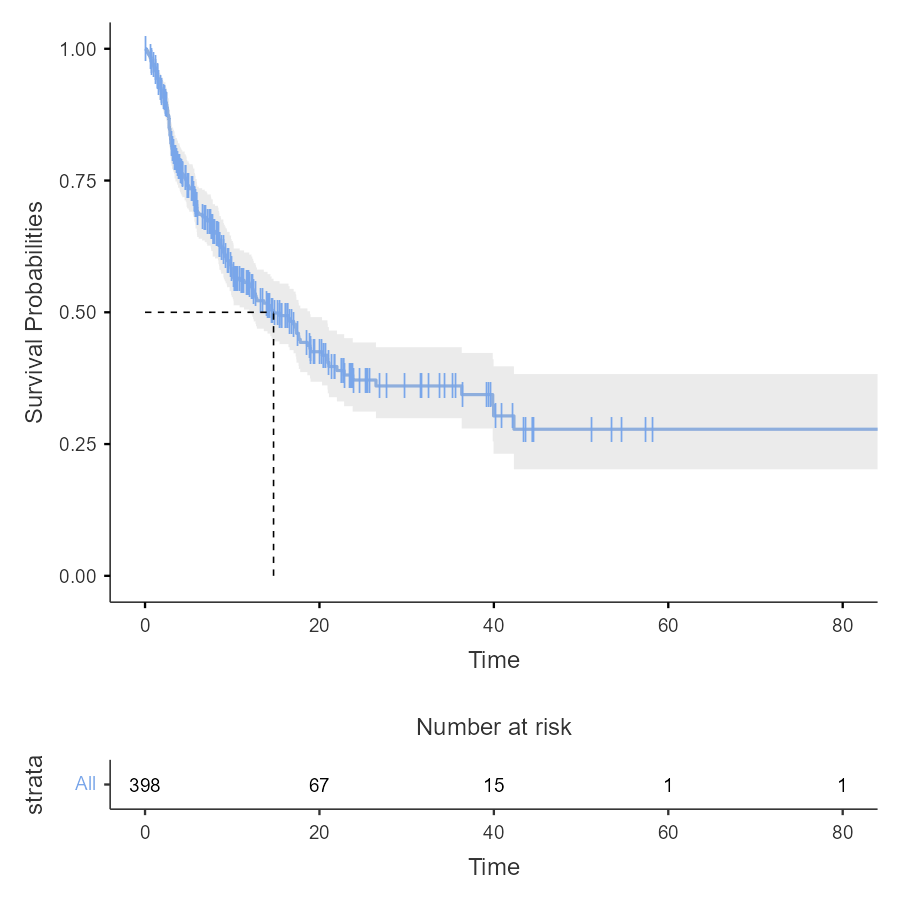


**Supplementary Fig. 1b** Survival curve based on Kaplan-Meier estimates of OS


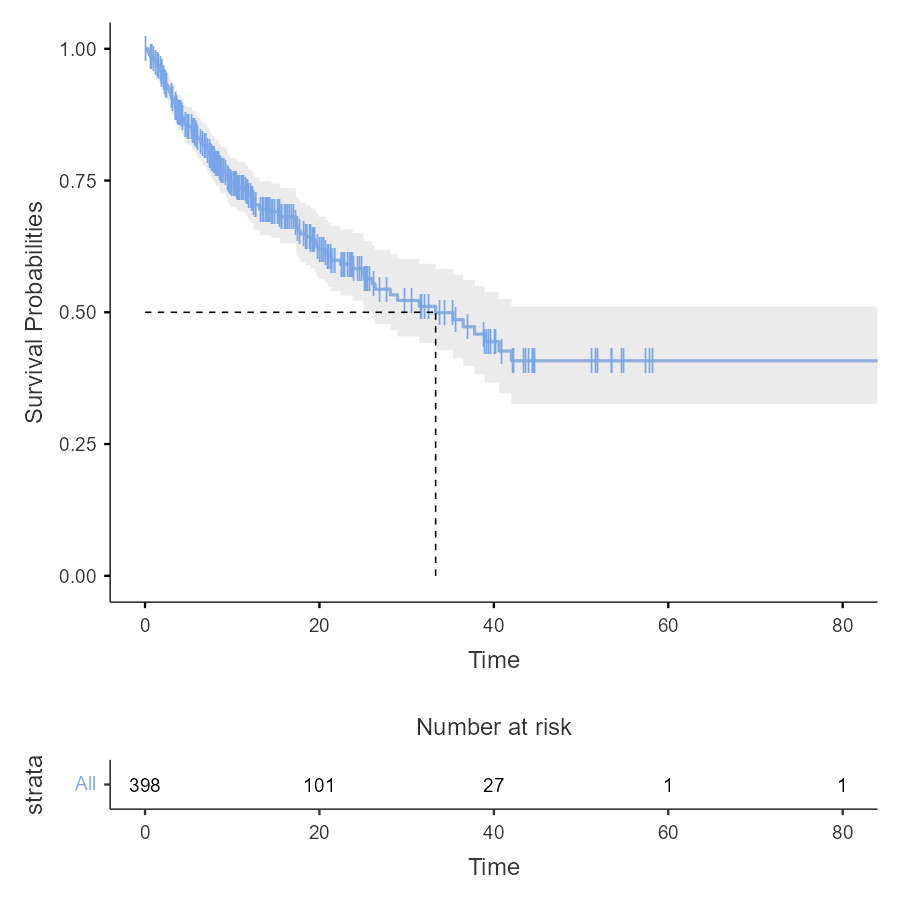


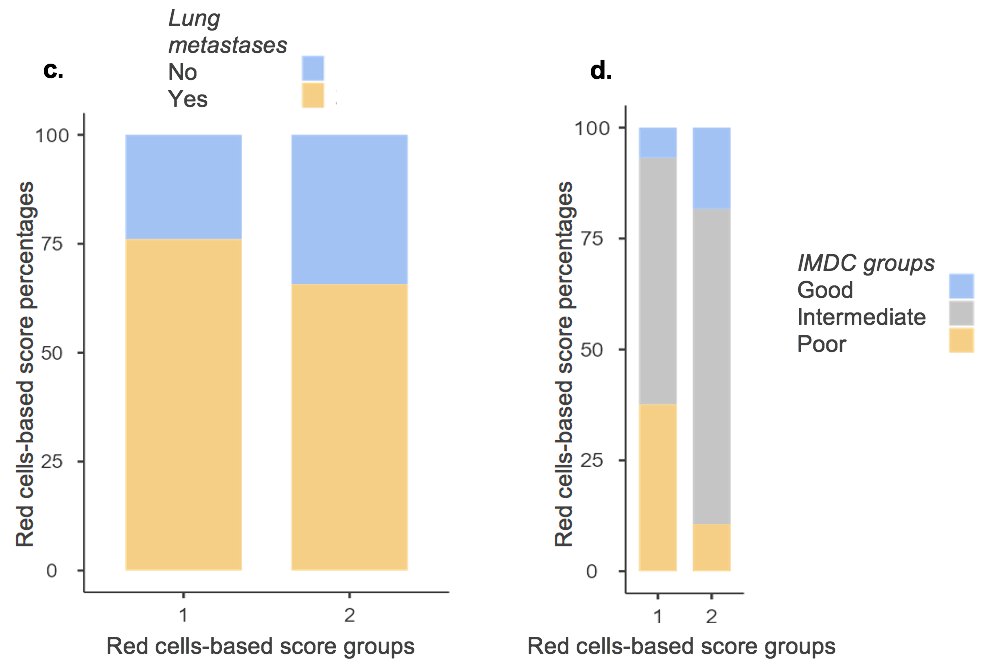

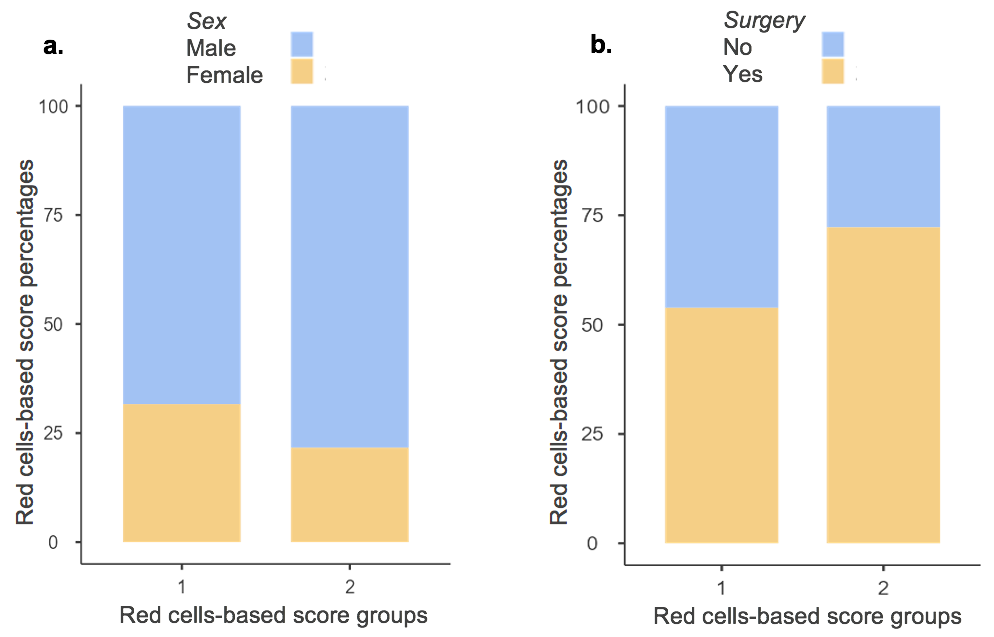


**Supplementary Fig. 2** Stacked bar charts illustrating the percentage of patients belonging to the two *red cell-based score* groups according to sex (**a**), nephrectomy (**b**), lung metastases (**c**) and IMDC risk score (**d**). Scatter plot documenting the distribution of BMI values among the two *red cell-based score* groups. Mean and SD BMI values are inscribed in red.

**1**: unfavourable group; **2**: favourable group;


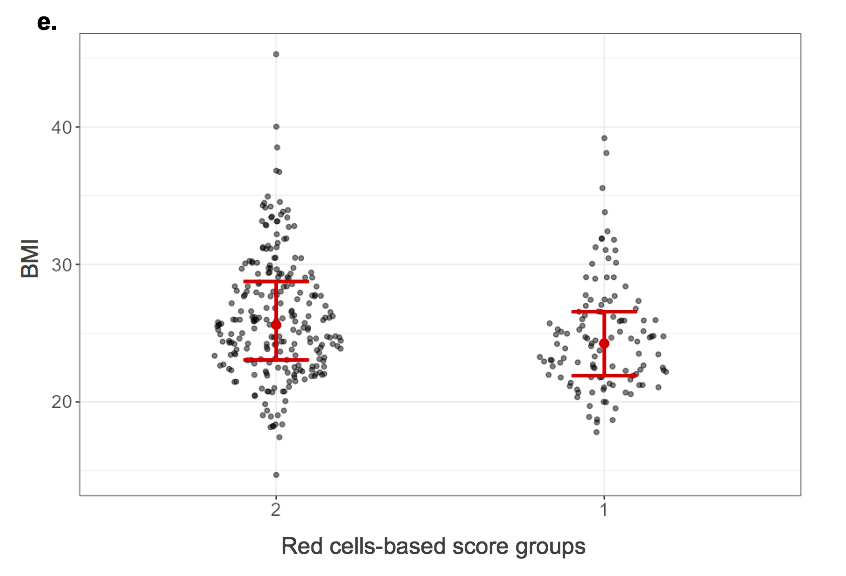


*Abbreviations. IMDC: International Metastatic RCC Database Consortium Score; BMI: body mass index.*


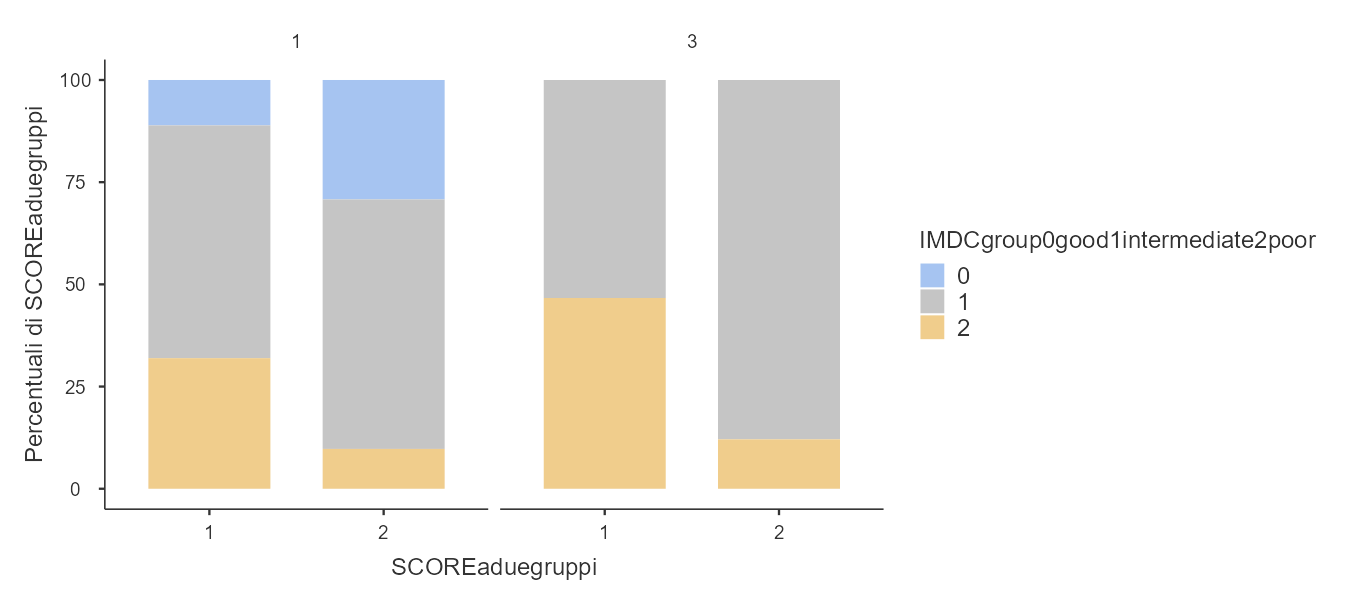

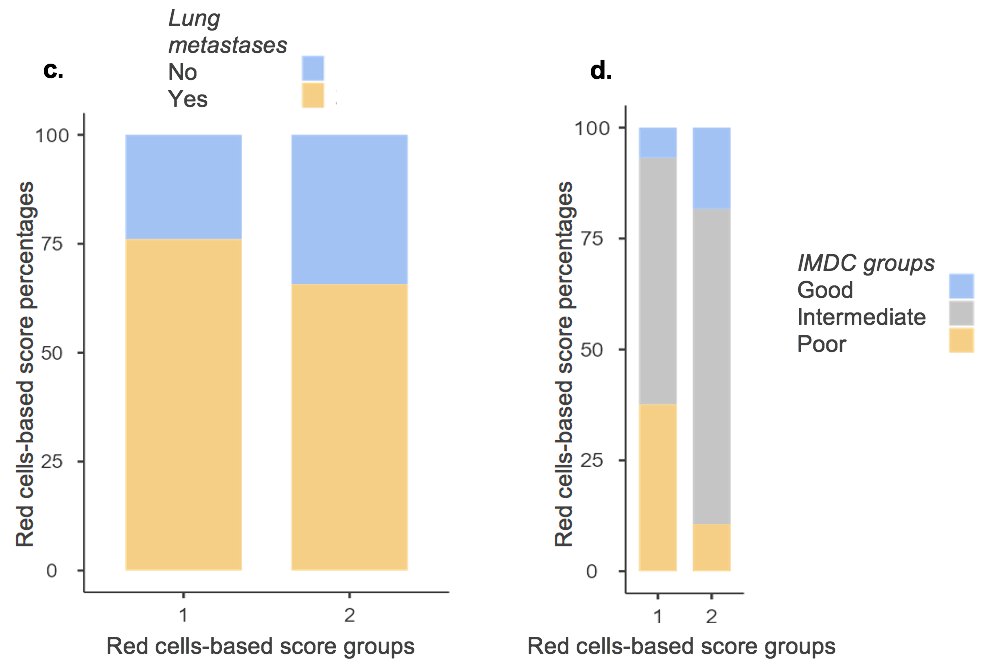


**TKI + ICI**

**ICI + ICI**


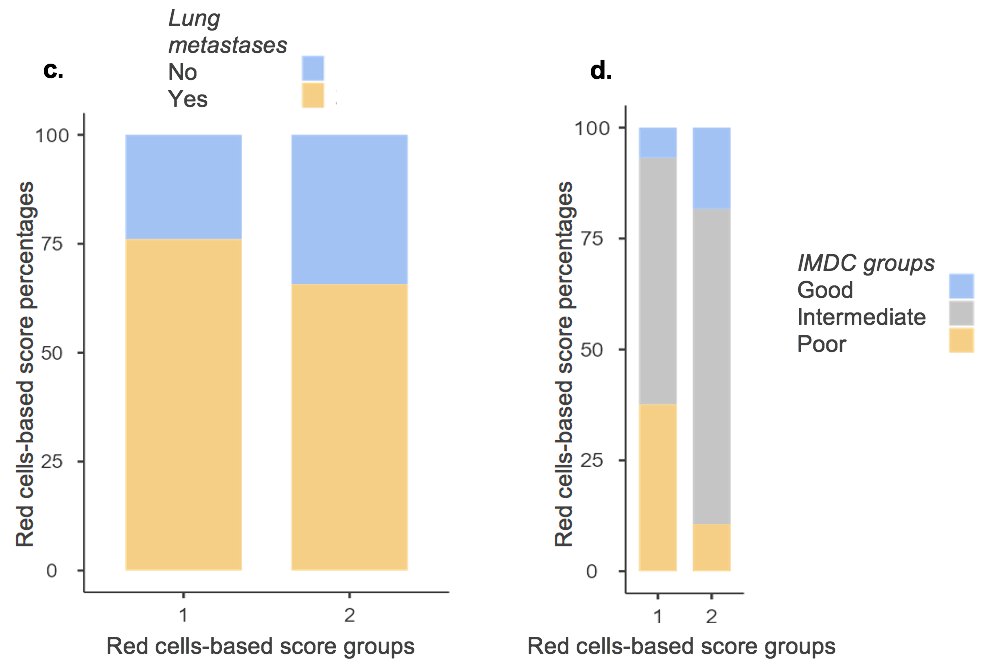


**Supplementary Fig. 3** Stacked bar graph illustrating the distribution of red cell-blood score groups among IMDC categories. **1**: unfavourable group; **2**: favourable group.

*Abbreviations. IMDC: International Metastatic RCC Database Consortium Score TKI: Tyrosine-Kinase Inhibitor; ICI: Immune Checkpoint Inhibitor.*

**Supplementary Table 2** Cross table of the IMDC categories distribution within the blood score groups. **Group 1**: unfavourable. **Group 2:** favourable.

| **Combination type** | **Red cell-based score** | **IMDC** | | | **Total** |
| --- | --- | --- | --- | --- | --- |
|  |  | Good | Intermediate | Poor |  |
| TKI + ICI | Group 1 | 8 | 41 | 23 | 72 |
|  | Group 2 | 45 | 94 | 15 | 154 |
|  | **total** | 53 | 135 | 38 | 226 |
| ICI + ICI | Group 1 | 0 | 24 | 21 | 45 |
|  | Group 2 | 0 | 80 | 11 | 91 |
|  | **total** | 0 | 104 | 32 | 136 |
| Total | Group 1 | 8 | 65 | 44 | 117 |
|  | Group 2 | 45 | 174 | 26 | 245 |
|  | **total** | 53 | 239 | 70 | 362 |

*Abbreviations. IMDC: International Metastatic RCC Database Consortium Score; TKI: Tyrosine-Kinase Inhibitor; ICI: Immune Checkpoint Inhibitor.*

**Supplementary Fig. 4** Hazard regression plot of PFS prognostic factors


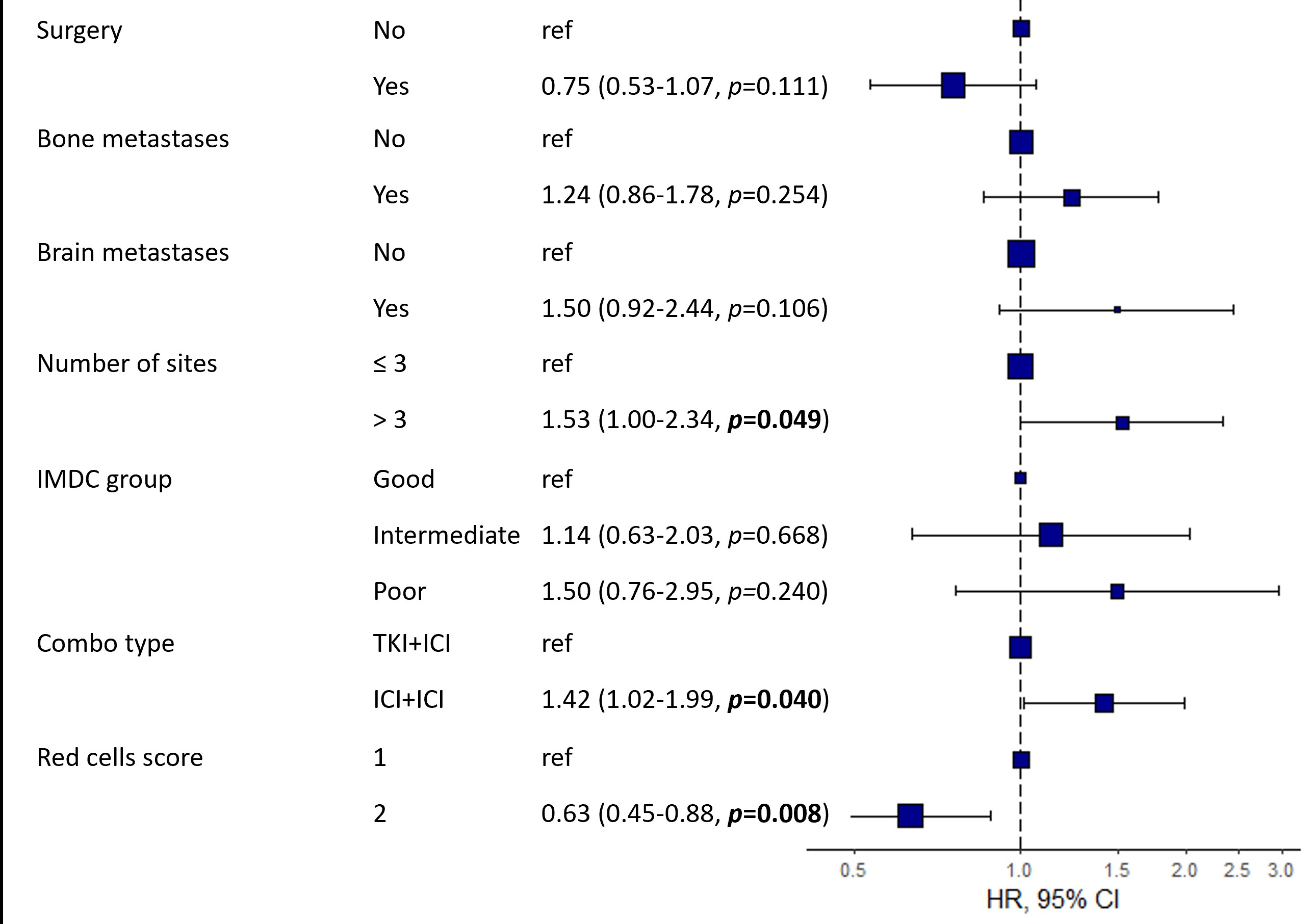


*Abbreviations. ref: reference; IMDC: International mRCC Database Consortium score; TKI: Tyrosine-Kinase Inhibitor; ICI: Immune Checkpoint Inhibitor.* ***1****: unfavourable group;* ***2****: favourable group.*

**Supplementary Fig. 5** Hazard regression plot of OS prognostic factors


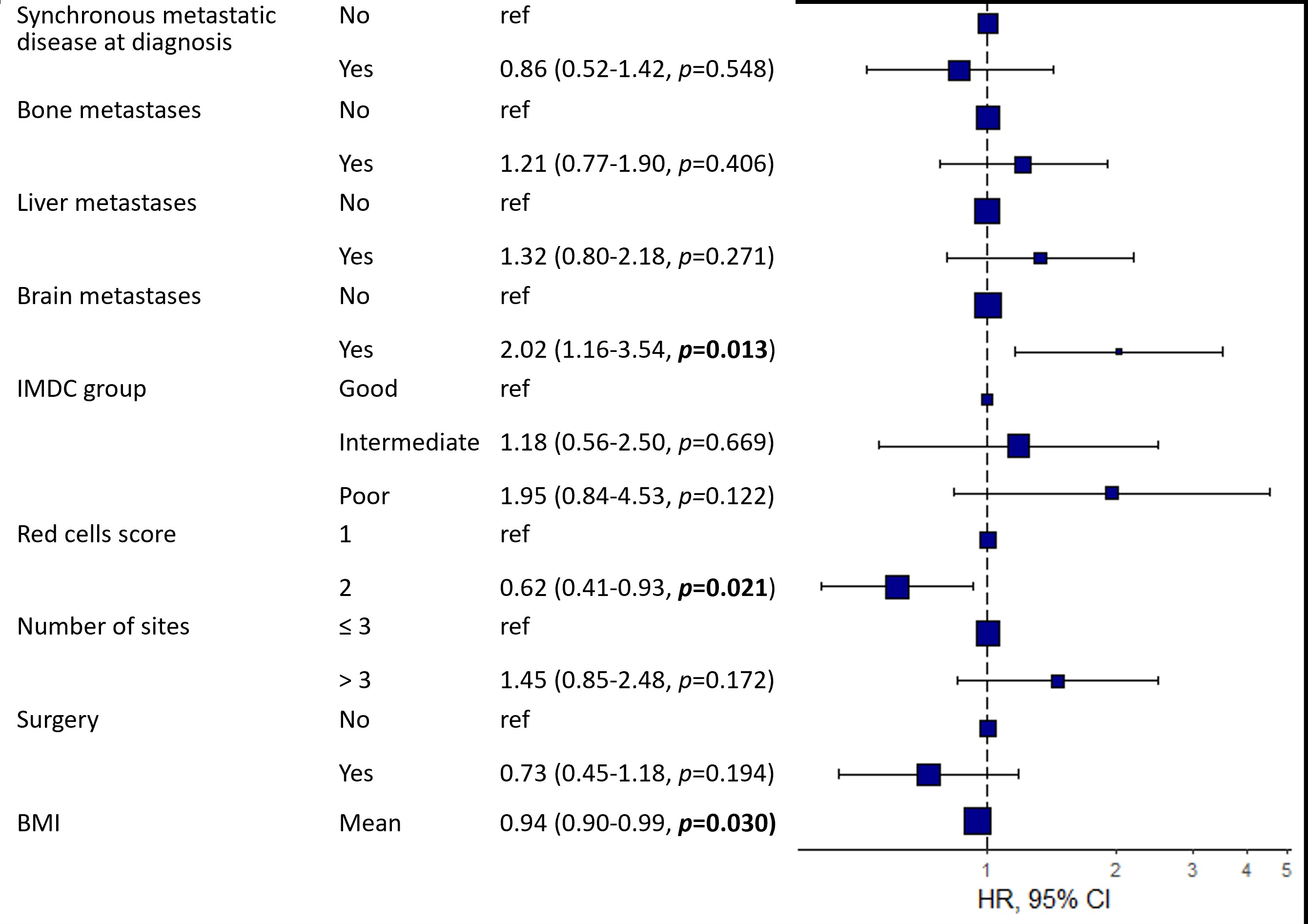


Abbreviations. ref: reference; IMDC: International mRCC Database Consortium score; TKI: Tyrosine-Kinase Inhibitor; ICI: Immune Checkpoint Inhibitor; BMI: Body Mass Index. **1**: unfavourable group; **2**: favourable group.

**Supplementary Fig. 6** Odds ratio regression plot of predictive factors


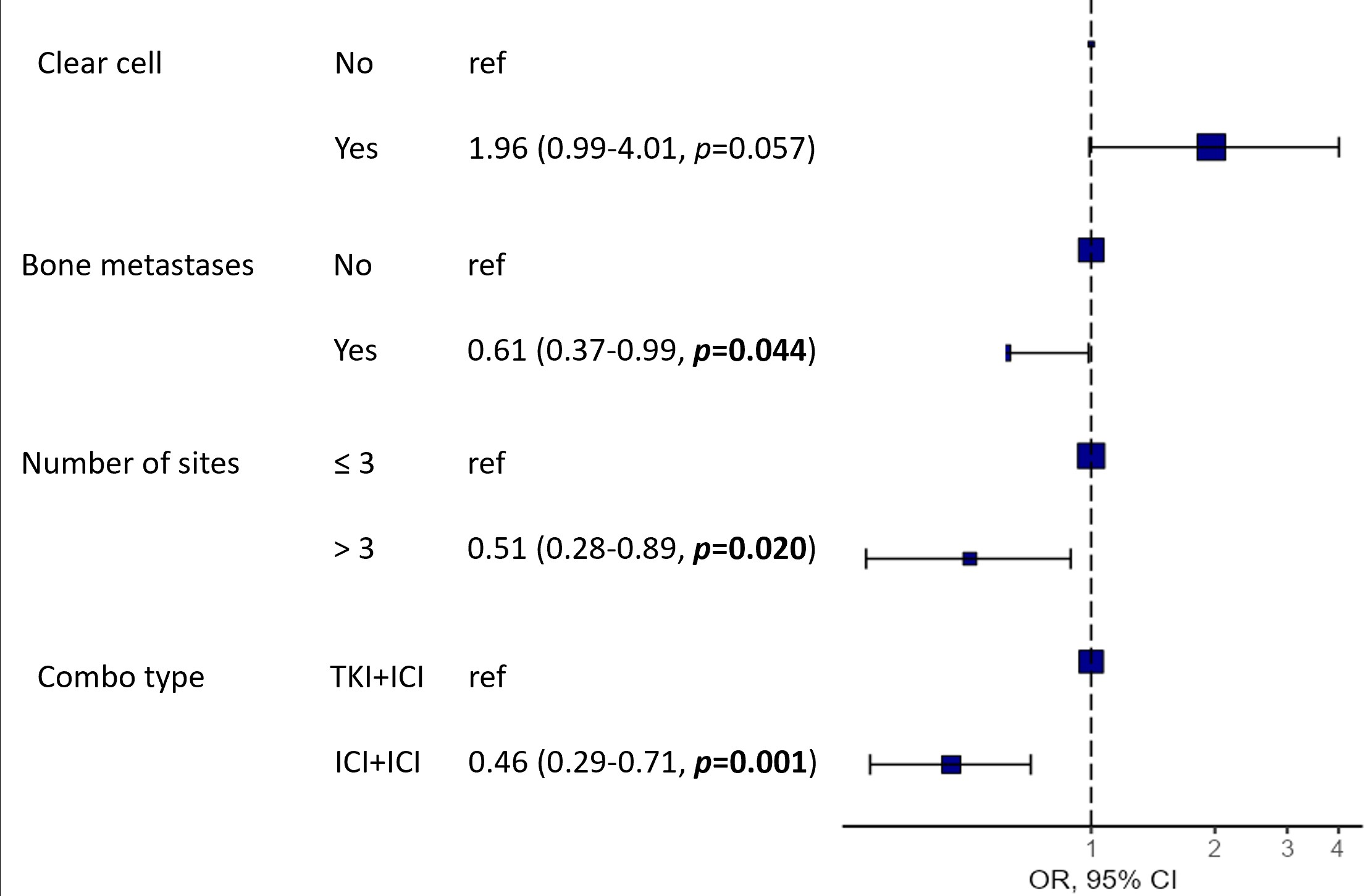


*Abbreviations. ref: reference; TKI: Tyrosine-Kinase Inhibitor; ICI: Immune Checkpoint Inhibitor.*

*.*

**Supplementary Fig. 7** Kaplan-Meier estimates of PFS according to therapy combination


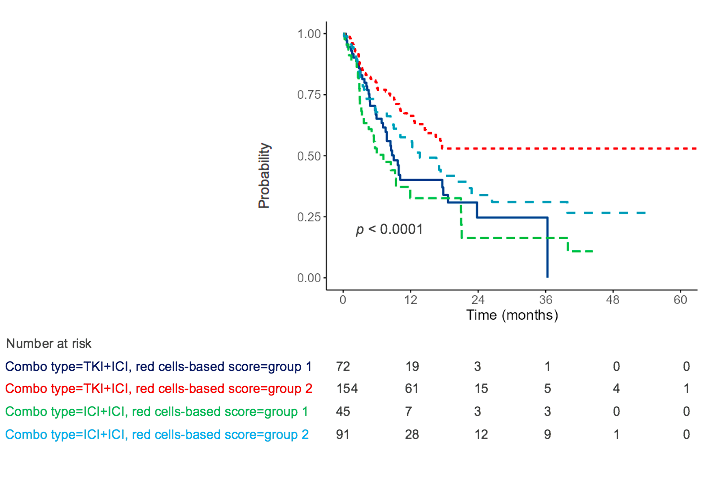


***Red cell-based score* group:**

Group 1: unfavourable

Group 2: favourable

**Combination type:**

TKI+ICI: Tyrosine Kinase Inhibitors plus Immune Checkpoint Inhibitors

ICI+ICI: ipilimumab plus nivolumab


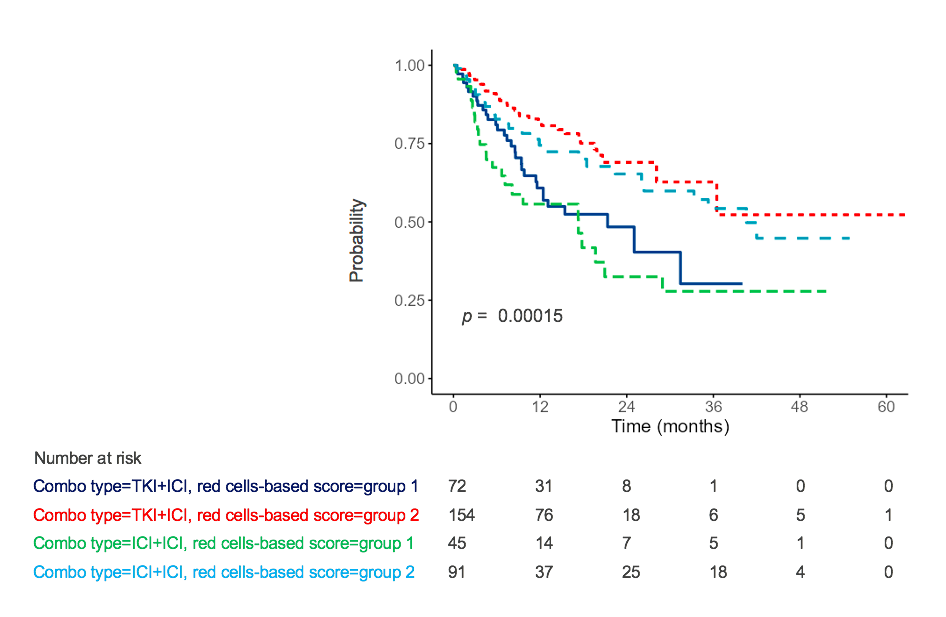


***Red cell-based score* group:**

Group 1: unfavourable

Group 2: favourable

**Combination type:**

TKI+ICI: Tyrosine Kinase Inhibitors plus Immune Checkpoint Inhibitors

ICI+ICI: ipilimumab plus nivolumab

**Supplementary Fig. 8** Kaplan-Meier estimates of OS curves according to therapy combination
